# Gray Matter Changes in the Insular Cortex During the Course of the Schizophrenia Spectrum

**DOI:** 10.3389/fpsyt.2020.00659

**Published:** 2020-07-10

**Authors:** Tsutomu Takahashi, Mikio Kido, Daiki Sasabayashi, Mihoko Nakamura, Atsushi Furuichi, Yoichiro Takayanagi, Kyo Noguchi, Michio Suzuki

**Affiliations:** ^1^Department of Neuropsychiatry, University of Toyama Graduate School of Medicine and Pharmaceutical Sciences, Toyama, Japan; ^2^Research Center for Idling Brain Science, University of Toyama, Toyama, Japan; ^3^Arisawabashi Hospital, Toyama, Japan; ^4^Department of Radiology, University of Toyama Graduate School of Medicine and Pharmaceutical Sciences, Toyama, Japan

**Keywords:** insular cortex, magnetic resonance imaging, schizophrenia, schizotypal disorder, progressive changes

## Abstract

Progressive gray matter reductions in the insular cortex have been reported in the early phases of schizophrenia (Sz); however, the trajectory of these reductions during the course of the illness currently remains unclear. Furthermore, it has not yet been established whether patients with schizotypal (SzTypal) features exhibit progressive changes in the insular cortex. This follow-up magnetic resonance imaging study examined volume changes in the short and long insular cortices (mean inter-scan interval = 2.6 years) of 23 first-episode (FE) and 17 chronic patients with Sz, 14 with SzTypal disorder, and 21 healthy controls. Baseline comparisons revealed smaller insular cortex volumes bilaterally in Sz patients (particularly in the chronic group) than in SzTypal patients and healthy controls. FESz patients showed significantly larger gray matter reductions in the insular cortex over time (left: −3.4%/year; right: −2.9%/year) than those in healthy controls (−0.1%/year for both hemispheres) without the effect of subregion or antipsychotic medication, whereas chronic Sz (left: −1.5%/year; right: −1.6%/year) and SzTypal (left: 0.5%/year; right: −0.6%/year) patients did not. Active atrophy of the right insular cortex during FE correlated with fewer improvements in positive symptoms in the Sz groups, while mild atrophy of the left insular cortex during the chronic phase was associated with the severity of negative symptoms in the follow-up period. The present results support dynamic volumetric changes in the insular cortex being specific to overt Sz among the spectrum disorders examined and their degree and role in symptomatology appear to differ across the illness stages.

## Introduction

Gray matter reductions in the insular cortex, particularly in the anterior portion (i.e., the short insula), which has emotional and language-related functions (1, 2), are one of the most definitive findings in schizophrenia (Sz) (3, 4). Further, previous functional neuroimaging evidences have suggested that abnormal activation and/or connectivity in the brain regions associated with the salience network, including the insular cortex, are involved in the production and persistence of auditory hallucinations (5–7). Morphological changes of the insular cortex may exist in the first episode (FE) or even prior to illness onset (3, 8, 9), supporting its early neurodevelopmental pathology. However, longitudinal magnetic resonance imaging (MRI) studies demonstrated progressive gray matter loss during the course of Sz, which appears to be non-linear and most prominent in the early illness stages (10, 11). Although dynamic brain alterations in peri-Sylvian regions (such as the superior temporal gyrus and insula) around the onset of Sz have been suggested to contribute to positive psychotic symptoms (12), it currently remains unclear whether mild atrophy at the later illness stage is still involved in clinical manifestations. Due to the lack of detailed medication data in these previous studies (10, 11), it also has not yet been established whether antipsychotic medication significantly influences insular gray matter changes during the course of the illness. Furthermore, limited information is currently available on whether the putative pathophysiological trajectory of the insular cortex is specific to overt Sz or commonly exists within spectrum disorders.

Patients with schizotypal (SzTypal) disorder (13) or SzTypal personality disorder (14), a prototypical Sz spectrum condition, have biological and phenomenological commonalities with more severely ill patients with Sz (15). They may exhibit similar gray matter reductions in the temporal regions with overt Sz, which may represent a common susceptibility to Sz, but show less prominent changes in the fronto-limbic regions (16–18). Although only a few longitudinal MRI studies have been conducted on SzTypal patients, they are also characterized by the absence of the progressive gray matter reductions observed in the temporal regions of Sz (19, 20), which supports the notion that the active brain pathological process underlying the emergence of overt psychosis does not occur in SzTypal patients (15). On the other hand, although our previous cross-sectional study found no gray matter reductions in the anterior or posterior somatosensory (i.e., long insula) portions (1, 2) of the insular cortex in SzTypal patients (21), it is unknown whether they exhibit insular gray matter changes over time.

This longitudinal MRI study was conducted to investigate the trajectory of gray matter changes in insular subregions in the Sz spectrum (i.e., Sz and SzTypal disorder patients). Volume changes in the short and long insular cortices were compared between patients with FESz, chronic Sz, SzTypal, and healthy controls. Due to the different phenomenology between Sz and SzTypal patients in addition to the potential role of a dynamic pathological brain process in the development of psychosis (18), we predicted progressive insular atrophy in Sz (particularly the FE group), but not in SzTypal patients, which may be associated with symptomatology. We also examined the influence of antipsychotic medication on longitudinal insular changes.

## Materials and Methods

### Participants

Study participants included 40 patients with Sz (23 FE and 17 chronic cases), 14 with SzTypal disorder, and 21 healthy controls (**Table 1**) who were scanned twice with an inter-scan interval of approximately 2 to 3 years using the same scanner/parameters; they were all right-handed and healthy physically without any previous history of serious medical diseases (including neurological illnesses and head trauma) or substance abuse disorder. The insular cortex volumes of 14/23 FESz, 4/17 chronic Sz, 12/14 SzTypal, and 14/21 healthy controls in this cohort were reported in our previous cross-sectional study (21); however, this was the first longitudinal study on the insular cortex using our data.

**Table 1:** Demographic/clinical characteristics of study participants.

	C	SzTypal	FESz	Chronic Sz	Group comparisons
	(12M, 9F)	(10M, 4F)	(15M, 8F)	(7M, 10F)	
Age (years) [range]	24.1 ± 5.6 [18.0 to 38.0]	23.0 ± 4.9 [16.3 to 34.4]	23.5 ± 4.8 [17.9 to 32.7]	31.2 ± 7.1 [19.4 to 45.3]	*F* (3, 71) = 8.14, *P* < 0.001; C, SzTypal, FESz < Chronic Sz
Height (cm)	165.0 ± 7.3	166.6 ± 9.2	165.0 ± 7.9	163.2 ± 9.2	*F* (3, 71) = 0.54, *P* = 0.660
Education (years)	15.1 ± 2.3	12.5 ± 2.4	13.1 ± 1.6	14.2 ± 2.5	*F* (3, 71) = 5.23, *P* = 0.003; SzTypal, FESz < C
Parental education (years)	12.8 ± 2.8	12.1 ± 1.6	12.7 ± 2.1	11.8 ± 1.5	*F* (3, 71) = 0.92, *P* = 0.438
Inter-scan interval (years) [range]	2.6 ± 0.4 [2.0 to 3.2]	2.9 ± 0.8 [1.8 to 4.4]	2.6 ± 0.8 [1.0 to 4.5]	2.2 ± 0.8 [1.2 to 4.0]	*F* (3, 71) = 2.60, *P* = 0.059
Onset age (years)	–	–	22.4 ± 4.8	22.7 ± 5.8	*F* (1, 38) = 0.03, *P* = 0.865
Illness duration at baseline (months) [range]	–	–	9.5 ± 9.1 [1.2 to 40.8]	96.2 ± 37.7 [43.2 to 168.0]	*F* (1, 38) = 114.59, *P* < 0.001; FESz < Chronic Sz
Duration of medication at baseline (months) [range]	–	42.2 ± 60.2 [1.2 to 204.0]	7.8 ± 9.7 [0 to 37.2]	75.9 ± 60.8 [1.2 to 196.8]	*F* (2, 51) = 10.56, *P* < 0.001; FESz < Chronic Sz
Medication type during follow-up (typical/atypical/mixed)	–	5/8/1	3/16/4	5/9/3	Fisher’s exact test, *P* = 0.49
Medication dose (HPD equivalent)					
Baseline (mg/day) [range]	–	6.4 ± 7.6 [1.2 to 29.5]	13.5 ± 11.4 [2.3 to 41.1]	10.9 ± 7.9 [2.7 to 33.1]	*F* (2, 51) = 2.48, *P* = 0.094
Cumulative dose during follow-up (mg) [range]	–	7030.7 ± 7243.2 [758.2 to 24303.0]	9526.4 ± 8609.2 [1333.0 to 37436.0]	11763.9 ± 11054.2 [849.1 to 43265.6]	*F* (2, 51) = 1.03, *P* = 0.365
Mean dose during follow-up (mg/day) [range]	–	6.2 ± 5.1 [0.9 to 17.1]	9.5 ± 7.2 [2.5 to 27.0]	15.1 ± 10.0 3.9 to 35.9]	*F* (2, 51) = 5.30, *P* = 0.008; SzTypal < Chronic Sz
SAPS total					
Baseline	–	17.6 ± 9.6 (*N*= 13)	29.0 ± 24.3 (*N*= 20)	31.9 ± 27.3 (*N*= 16)	*F* (2, 46) = 1.59, *P* = 0.216
Follow-up	–	13.6 ± 11.3 (*N*= 14)	17.0 ± 17.1 (*N*= 22)	33.7 ± 29.4 (*N*= 17)	*F* (2, 50) = 4.46, *P* = 0.016; SzTypal < Chronic Sz
SANS total					
Baseline	–	54.8 ± 22.1 (*N*= 13)	52.1 ± 25.5 (*N* = 20)	50.8 ± 16.4 (*N*= 16)	*F* (2, 46) = 1.21, *P* = 0.886
Follow-up	–	42.3 ± 16.6 (*N*= 14)	38.0 ± 22.5 (*N*= 22)	55.8 ± 19.1 (*N*= 17)	*F* (2, 50) = 3.96, *P* = 0.025; FESz < Chronic Sz

Values represent means ± SD unless otherwise stated. Groups were matched for gender (Chi-squared = 3.51, P = 0.319).

C, controls; F, female; FE, First-episode; HPD, haloperidol; M, male; SANS, Scale for the Assessment of Negative Symptoms; SAPS, Scale for the Assessment of Positive Symptoms. Sz, schizophrenia; SzTypal, schizotypal disorder.

As described elsewhere (19, 22), Sz and SzTypal patients fulfilling the research criteria for ICD-10 (13) were recruited from the in- and outpatient clinics of the Department of Neuropsychiatry, Toyama University Hospital. Diagnoses were confirmed in a structured clinical interview [i.e., the Comprehensive Assessment of Symptoms and History (23)], supplemented by a detailed review of medical charts and clinical symptoms, which were rated at the time of scanning using the Scale for the Assessment of Negative/Positive Symptoms [SANS/SAPS (24)]. FESz patients were defined by an illness duration ≤ 1 year (*N* = 19) or under first psychiatric hospitalization (*N* = 4) at baseline (25, 26), while chronic Sz patients were defined as those with an illness duration > 3 years at baseline. The sample characteristics and recruitment strategy of our clinic-based SzTypal cohort were previously described in detail (19, 22, 27, 28); participants also met the DSM Axis II diagnosis of SzTypal personality disorder (14) and none developed overt psychosis during the follow-up period (mean = 2.9 years, SD = 0.8). They visited our hospital because of the distress or associated problems (e.g., irritability, anxiety and depressive symptoms, and suicidal ideation) stemming from their SzTypal features, which required clinical care including antipsychotic medications. Thus, our SzTypal cohort was considered to be more severely ill than SzTypal individuals among the general population. **Table 1** shows a summary of the medication status of participants as well as other clinical data.

Healthy controls, who were screened for a personal or family history of neuropsychiatric disorders among first-degree relatives (29), were recruited from the community, university students, and hospital staff. The Committee on the Medical Ethics of Toyama University approved the study protocol. All study participants provided written informed consent in accordance with the Declaration of Helsinki. When participants were under the age of 20 years, written consent was also obtained from the parent or guardian.

### MRI Scanning

Participants were scanned twice at Toyama University Hospital by a 1.5T Magnetom Vision (Siemens Medical System, Inc., Erlangen, Germany) with identical protocols; T1-weighted 1.0-mm continuous sagittal images were obtained using three-dimensional gradient-echo sequence FLASH (time to echo = 5 ms, time repetition = 24 ms, flip angle = 40°, matrix size = 256 × 256, and voxel dimensions = 1 × 1 × 1 mm). As described previously (30), intracranial volume (ICV) was measured on reformatted 5-mm sagittal slices using the anatomical landmarks described by Eritaia et al. (31).

### Insular Cortex Measurements

**Figure 1:**
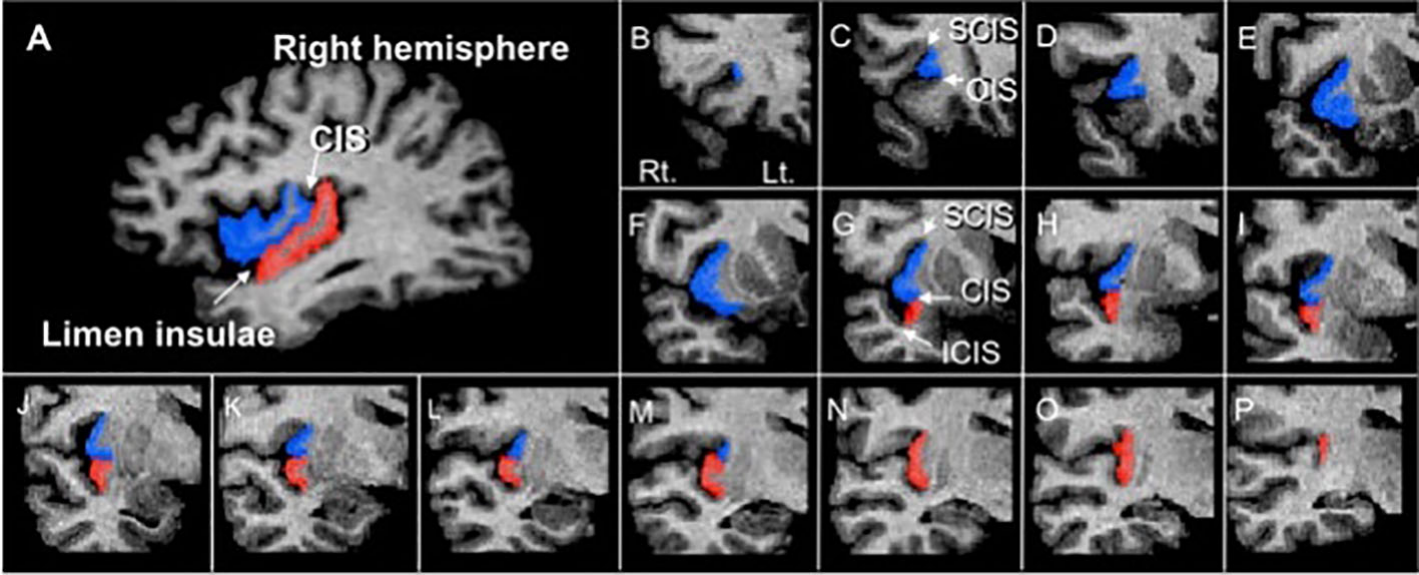
A sagittal slice **(A)** and sample coronal slices **(B–P)** of short (blue) and long (red) insular cortices manually traced in this study. CIS, central insular sulcus; ICIS, inferior circular insular sulcus; OIS, orbitoinsular sulcus; SCIS, superior circular insular sulcus.

The insular cortex was manually measured on reconstructed 1-mm continuous coronal slices of the gray matter component, which was automatically segmented by the signal intensity histogram distribution of the whole cerebrum, using Dr. View software (Infocom, Tokyo, Japan) (32). As fully described previously (21, 33), the short (anterior) and long (posterior) insular cortices were manually delineated by one rater (TT) blinded to the identity of participants and the time at which scanning was performed (baseline or follow-up). Specific anatomical landmarks for measurements (i.e., the orbito- and central insular sulci, superior and inferior circular insular sulci, and limen insulae) were easily identified using sagittal and coronal views (**Figure 1**). The inter- (TT and MK) and intra-rater intraclass correlation coefficients (ICC) of the measurements in 10 randomly selected brains were all > 0.87.

### Statistical Analysis

A one-way analysis of variance (ANOVA), the chi-squared test, and Fisher’s exact test were performed to assess group differences in demographic and clinical data.

In cross-sectional comparisons of insular cortex volumes, absolute volume was analyzed using an analysis of covariance (ANCOVA) with the between-subject factor of diagnosis, within-subject variables of hemisphere and subregion (short, long), and covariates of age, ICV, and medication dose. Regarding longitudinal volume changes in the insular cortices, the dependent variable % volume change [100 × (absolute volume at the follow-up - absolute volume at the baseline)/absolute volume at the baseline] was used; ANCOVA was performed using the between-subject factor of diagnosis, within-subject variables of hemisphere and subregion, and covariates of age at baseline, ICV, inter-scan interval, and cumulative dose of antipsychotics during scans. Post-hoc Spjotvoll & Stoline tests were used to follow up significant effects yielded in ANCOVA. The insular volumes used in these ANCOVAs (i.e., absolute volume and % volume change) were normally distributed (tested by the Shapiro-Wilk test). The statistical conclusions reported here did not change even when absolute volume changes in the insular cortex over time were assessed by ANCOVA with time (baseline, follow-up) as the within-subject variable, when gender was added as a covariate, when the covariates without significant group differences were excluded, or when only 19 patients with Sz with illness duration ≤ 1 year were included in the FESz group.

Relationships between % volume changes in the insular cortex and clinical variables [total SANS/SAPS scores (score changes between scans and scores at second scan) and cumulative medication doses during the follow-up] were investigated by Pearson’s partial correlation coefficients controlled for the inter-scan interval and ICV. SAPS scores and medication doses, which did not have a normal distribution (tested by the Shapiro-Wilk test), were log-transformed. The insular volume was not subdivided into the short and long cortices because of the lack of prominent subregional effects for its longitudinal changes. To reduce the Type I error rate, relationships were tested based on previous hypotheses. Since dynamic brain changes around the onset of Sz may be associated with the development and treatment responses of positive and negative symptoms (11, 12), we examined the relationship between % volume changes in the insular cortex and SANS/SAPS score changes in the FESz group. Due to the lack of active symptom changes in the chronic stage (**Table 1**) as well as the notion that prolonged brain changes may lead to poor functional outcomes and severely negative symptoms (12, 34), the potential relationship between % volume changes in the insular cortex and total SANS scores in the follow-up was examined in chronic Sz patients. SzTypal patients were excluded from correlational analyses because of the lack of longitudinal insular changes. The results of these correlational analyses remained essentially the same even when we used no controlling factors (i.e., inter-scan interval and ICV).

A *P*-value of < 0.05 was considered to be significant. A Bonferroni correction was employed for correlation analyses.

## Results

### Sample Characteristics

Groups in the present study were matched for gender, height, parental education, and the inter-scan interval, while the education level was lower in the FESz and SzTypal groups than in the control group. As expected, the chronic Sz group was characterized by an older age than all other groups, a longer illness/medication duration than the FESz group, and more severe symptoms than the other clinical groups (**Table 1**).

### Cross-Sectional Comparisons of the Insular Cortex

**Table 2** shows baseline comparisons, which revealed a smaller insular cortex in the FE and chronic Sz groups than in the SzTypal (post-hoc tests, *vs.* FESz and chronic Sz: *P* < 0.001) and control (post-hoc tests, *vs.* FESz: *P* = 0.035; *vs.* chronic Sz: *P* < 0.001) groups. The insular cortex was also significantly smaller in the chronic Sz group than in the FESz group (post-hoc test, *P* = 0.004). No significant group-by-subregion (**Table 2**) and group-by-hemisphere [*F* (3, 71) = 2.69, *p* = 0.053] interactions were observed.

**Table 2:** Brain measurements of study participants.

	C	SzTypal	FESz	Chronic Sz	Effect of diagnosis	Diagnosis × subregion
Intracranial volume (cm^3^)	1495 ± 138	1611 ± 119	1476 ± 140	1527 ± 194	*F* (3, 70) = 2.84, *P* = 0.044^a^	–
Whole gray matter (cm^3^)						
Baseline	698 ± 68	750 ± 73	685 ± 78	653 ± 69	*F* (3, 68) = 1.69, *P* = 0.177	–
Follow-up	694 ± 76	733 ± 59	661 ± 65	639 ± 71	*F* (3, 68) = 1.49, *P* = 0.225	–
% change/year	-0.3 ± 1.2	-0.8 ± 2.0	-1.2 ± 2.0	-1.2 ± 2.4	*F* (3, 67) = 0.88, *P* = 0.459	–
Whole insular cortex						
Baseline (mm^3^)					*F* (3, 68) = 8.53, *P* < 0.001; Chronic Sz < FESz <C, SzTypal	*F* (3, 71) = 1.50, *P* = 0.223
L	8317 ± 784	8536 ± 970	7668 ± 1113	6723 ± 1003		
R	7859 ± 735	8566 ± 1015	7380 ± 896	6723 ± 906		
Follow-up (mm^3^)					*F* (3, 68) = 9.26, *P* < 0.001; Chronic Sz, FESz < C, SzTypal	*F* (3, 71) = 2.49, *P* = 0.067
L	8324 ± 911	8643 ± 991	6999 ± 1116	6508 ± 1005		
R	7834 ± 768	8409 ± 850	6835 ± 913	6537 ± 936		
% change/year^b^					*F* (3, 67) = 7.55, *P* < 0.001; FESz < Chronic Sz, C, SzTypal	*F* (3, 71) = 0.89, *P* = 0.453
L	-0.1 ± 1.3	0.5 ± 1.4	-3.4 ± 3.7	-1.5 ± 2.2		
R	-0.1 ± 1.4	-0.6 ± 1.4	-2.9 ± 2.7	-1.6 ± 2.5		
Short insular cortex						
Baseline (mm^3^)					–	–
L	5478 ± 690	5424 ± 661	5051 ± 688	4365 ± 771		
R	5025 ± 612	5505 ± 642	4777 ± 713	4342 ± 763		
Follow-up (mm^3^)					–	–
L	5502 ± 790	5437 ± 648	4633 ± 674	4214 ± 700		
R	5017 ± 696	5434 ± 602	4444 ± 667	4196 ± 799		
% change/year^b^					–	–
L	0.0 ± 1.9	0.2 ± 1.8	-3.3 ± 3.3	-1.5 ± 2.2		
R	-0.1 ± 1.9	-0.4 ± 1.2	-2.8 ± 2.6	-1.9 ± 2.6		
Long insular cortex						
Baseline (mm^3^)					–	–
L	2839 ± 532	3112 ± 627	2617 ± 650	2357 ± 593		
R	2834 ± 516	3061 ± 666	2603 ± 496	2381 ± 445		
Follow-up (mm^3^)					–	–
L	2822 ± 526	3206 ± 641	2366 ± 645	2294 ± 614		
R	2817 ± 460	2975 ± 593	2391 ± 468	2342 ± 430		
% change/year^b^					–	–
L	-0.2 ± 2.6	1.1 ± 1.6	-3.7 ± 5.0	-1.3 ± 2.2		
R	-0.1 ± 2.1	-0.8 ± 2.6	-3.2 ± 3.3	-1.1 ± 2.7		

Values represent means ± SD.C, controls; FE, First-episode; L, left; R, right; Sz, schizophrenia; SzTypal, schizotypal disorder.^a^Age was used as a covariate. Post-hoc tests showed no significant group differences.^b^A negative value indicates a decrease in volume. Statistical analyses were based on % changes controlling for inter-scan intervals (see text).

Similar results were obtained in group comparisons in follow-ups, with a smaller insular volume being observed in the FE and chronic Sz groups than in the SzTypal and control groups (post-hoc tests, all *P* < 0.001) and no prominent subregional effect (**Table 2**). However, no significant differences were observed in the insular cortex volume between the FE and chronic Sz groups in follow-ups (post-hoc test, *P* = 0.278).

### Longitudinal Comparison of the Insular Cortex

**Figure 2:**
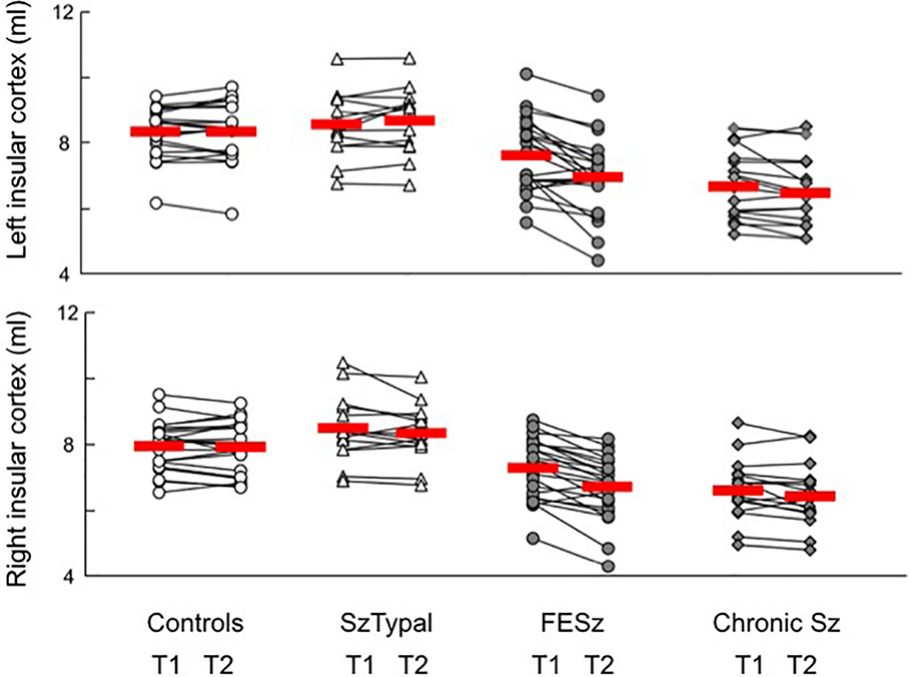
Absolute volume of the insular cortex in participants. Due to the absence of the significant effect of subregion (i.e., short and long insular cortices), we showed the volumes of the whole insular cortex. The values of baseline (T1) and follow-up (T2) scans in each participant are connected with a straight line. Horizontal bars indicate the means of each group.

Volume reductions over time were significantly larger in the FESz group than in all other groups (post-hoc tests, *vs.* chronic Sz: *P* = 0.009; *vs.* SzTypal and controls: *P* < 0.001) (**Figure 2**), with no significant group-by-subregion (**Table 2**) and group-by-hemisphere [*F* (3, 71) = 1.99, *p* = 0.123] interactions.

We also tested the potential effect of the medication type during follow-ups [atypical (*N* = 16) vs. typical or mixed (*N* = 7)] on progressive insular atrophy in the FESz group, but did not find any effect.

### Correlational Analyses

**Figure 3:**
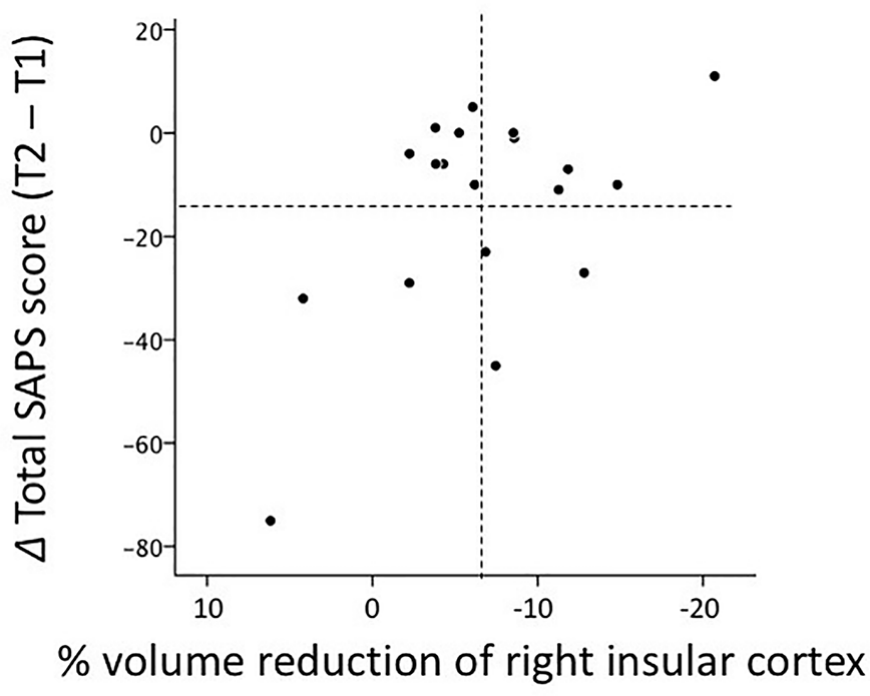
Relationship between gray matter reductions in the right insular cortex over time and score changes in Scale for the Assessment of Positive Symptoms (SAPS) between baseline (T1) and follow-up (T2) scans (i.e., absolute total score at T2 - absolute total score at T1) in 19 patients with first-episode schizophrenia. Regarding % volume reductions, negative values indicate volume decreases. Dotted lines indicate the mean values (all values in the figure are within three standard deviations of the mean).

Greater volume reductions in the left (*r* = 0.532, *P* = 0.028) and right (*r* = 0.596, *P* = 0.012) insular cortices over time in the FESz group correlated with fewer improvements (or the deterioration) in positive symptoms rated by total SAPS scores, which survived the Bonferroni correction for multiple comparisons for the right hemisphere (**Figure 3**). This correlation was not found for changes in total SANS scores (left, *r* = 0.204, *P* = 0.432; right, *r* = 0.097, *P* = 0.712).

In follow-ups on chronic Sz, volume reductions over time in the insular cortex correlated with higher total SANS scores on the left (*r* = 0.602, *P* = 0.018), but not right (*r* = 0.373, *P* = 0.171), hemisphere.

In the FE and chronic Sz groups, insular cortex volume changes did not correlate with cumulative medication doses between scans.

We also investigated whether a relationship exists between % gray matter reductions in the insular cortex and superior temporal gyrus (19) in FESz (*N* = 18), but found no correlation (left, *r* = −0.245, *P* = 0.360; right, *r* = 0.215, *P* = 0.424).

## Discussion

As far as we know, this is the first follow-up MRI study that investigated progressive gray matter changes in insular subregions at different stages of Sz (i.e., the FE and chronic phases) as well as in SzTypal disorder. The results obtained revealed significant longitudinal insular atrophy over time in the FESz group without prominent subregional or medication effects, which was associated with treatment responses to positive psychotic symptoms. We also detected mild (non-significant) insular atrophy in chronic Sz that might contribute to the severity of negative symptoms. In contrast, no insular volume changes were observed in SzTypal patients or the healthy controls during the follow-up period. These results support the dynamic pathophysiological trajectory of the insular cortex being specific to overt Sz among the spectrum disorders examined and also suggest that its degree and role in symptomatology may differ across the illness stages.

The present results obtained on dynamic gray matter atrophy during the course of Sz are consistent with the findings of a longitudinal MRI investigation on an independent cohort at various stages of psychosis, in which patients with psychotic disorders (predominantly Sz) had a smaller insular volume even before illness onset, but exhibited further active gray matter reductions during the transition period (−5.0%/year) and thereafter (−2.2%/year), followed by mild atrophy over time in the chronic stages (−0.7%/year) (10, 11). Consistent with previous findings, we found that patients with greater insular gray matter loss during FE showed fewer improvements in or the deterioration of clinical symptoms (10, 11, 35), particularly positive psychotic symptoms, and also demonstrated that mild but prolonged atrophy at the later illness stages may still contribute to negative symptomatology. These results support the notion that “late neurodevelopmental” pathologies around illness onset may underlie the development of psychosis (36, 37) and that progressive brain changes, which may differ between the clinical subtypes (38), may persist even at the later illness stages at least in specific subgroups of Sz and cause continued clinical deterioration (12, 34). Furthermore, while the trajectory of insular changes and its relationship with psychotic symptoms in the early stages are consistent with previous findings obtained on the superior temporal gyrus (19, 39, 40), the lack of a direct relationship between longitudinal changes in these peri-Sylvian structures suggests regional specificity in dynamic brain changes of Sz.

In spite of the results obtained in this structural MRI study, the mechanisms underlying insular gray matter changes in Sz remain unclear. However, a reduced insular volume before or at the onset of psychosis (3, 8, 9), particularly for the short insula (4, 10), may be partly explained by a post-mortem histological finding suggestive of neuronal migratory disturbances during early neurodevelopment in this region (41). On the other hand, potential mechanisms for the ongoing insular atrophy observed may include the excessive elimination of synapses, anomalies in synaptic plasticity, and neurotoxic effects caused by a glutamatergic excess due to *N*-methyl-*D*-aspartate receptor hypofunction (36, 42). The dynamic brain pathology in Sz may (19, 43) or may not (44, 45) be ameliorated by antipsychotic medication; however, the present results showed that medication did not significantly affect insular gray matter changes. The findings of a previous cross-sectional MRI study on medication-naïve FESz also supported age-related insular atrophy being independent of antipsychotic medication (42). Nevertheless, further longitudinal studies ideally using multi-modal techniques are needed to obtain a more detailed understanding of the longitudinal pathologies of Sz as well as the factors influencing these processes.

An important result of the present study was the absence of progressive gray matter changes in the insular cortex of SzTypal patients. In combination with previous cross-sectional findings showing that SzTypal patients are characterized by normal or even larger fronto-limbic structures (including the insular cortex) compared to healthy controls (18, 21, 22), the present results on SzTypal represent a potential protective factor against the overt psychosis and severe cognitive/social deficits associated with Sz (15). While the cross-sectional findings of common gray matter reductions in temporal regions (e.g., superior temporal gyrus) may underlie biological and phenomenological commonalities within the Sz spectrum (16–18), SzTypal patients do not appear to exhibit dynamic brain changes even in these temporal regions (19, 20). A recent follow-up MRI study showing the absence of progressive gray matter changes in the insular cortex in FE affective psychosis (35) also supports active pathological processes in the insular cortex being specific to overt Sz.

While this structural MRI study cannot directly address functional role of the insular abnormalities in the pathophysiology of Sz, previous functional neuroimaging studies have suggested that the insular cortex (especially its anterior portion) is involved in the generation of inner speech as a part of the salience network and is aberrantly active during the auditory hallucinations in Sz (5, 46). Interestingly, alterations of activation and functional connectivity in salience networks likely exist at earliest stages of Sz (47, 48) and are associated with treatment response (6), with the progression during early illness stages (47). Although the trajectory of salience network deficits at later illness stages and their contribution to negative symptomatology remain unclear, our longitudinal findings of the insular volume in SzTypal patients, who are generally spared from overt psychosis such as hallucinations (15), and different stages of Sz may partly support the concept of salience network dysfunction in Sz (5).

Several potential confounding factors need to be considered. First, the present preliminary study was clearly limited by the small sample size examined. Despite the potential role of the subregional specificity of insular abnormalities (i.e., anterior cognitive/affective *vs.* posterior somatosensory portions) in the pathophysiology of Sz (4, 49) as well as detailed manual delineation that could reflect inter-individual morphological variations of the insular subregions (1, 2), we failed to identify a subregional effect in cross-sectional and longitudinal insular findings. Furthermore, although typical and atypical antipsychotics may exert different effects on brain morphology in the early stages of Sz (50), we did not observe any effects of the antipsychotic types on insular volume changes over time. These somewhat unexpected results may be partly explained by the limited statistical power caused by the small sample size. It should be also noted that potential contribution of prolonged brain changes to clinical deterioration needs to be tested in future using larger cohorts of chronic Sz. Another limitation of sampling is that we could not exclude the possibility of some sampling biases. Indeed, at follow-up, FESz patients exhibited lower SAPS scores than those of chronic patients (**Table 1**), raising the possibility that only the patients with good treatment response have been successfully followed up and participated in this longitudinal study. Second, we did not systematically assess cognitive or social functioning in the patient groups. Based on the role of the insular cortex in social/emotional processing and various cognitive functions as a component of the “limbic integration cortex” (1, 2), the potential contributions of dynamic insular changes on socio-cognitive measures in Sz warrant further study. Finally, although we found no relationship between progressive changes in the insular cortex and another peri-Sylvian structure [i.e., superior temporal gyrus (19)] in the FESz group, other key brain regions [e.g., prefrontal cortex (51)] need to be investigated in future studies in order to clarify the regional specificity of the present results. Especially, future whole brain automated assessment would complement our hypothesis-driven region-of-interest findings.

In summary, the results of this follow-up MRI study revealed non-linear gray matter reductions in the insular cortex during the course of Sz that were more prominent during the early phases, but persisted even at the chronic stage and were not affected by antipsychotic medication. These progressive brain changes at various illness stages differently contributed to symptom formation in Sz, but were not observed in SzTypal patients, who were spared from developing overt psychosis. These results may reflect a pathophysiological trajectory that is specific to overt Sz among the spectrum disorders tested.

## Data Availability Statement

The datasets presented in this article are not readily available because we do not have permission to share the data. Requests to access the datasets should be directed to TT, tsutomu@med.u-toyama.ac.jp.

## Ethics Statement

The studies involving human participants were reviewed and approved by The Committee on the Medical Ethics of Toyama University. The patients/participants provided their written informed consent to participate in this study.

## Author Contributions

TT and MS conceived the concept and methods for the present study. TT performed statistical analyses and wrote the manuscript. DS, MN, and AF recruited participants and conducted clinical and diagnostic assessments. TT and MK analyzed MRI data. KN provided technical support for MRI scanning and data processing. AF, DS, and MN managed MRI and clinical data. YT and MS contributed to the writing and editing of the manuscript. All authors contributed to the article and approved the submitted version.

## Funding

This work was supported in part by JSPS KAKENHI Grant Number Nos. JP18K07550 to TT, JP18K15509 to DS, and by Health and Labour Sciences Research Grants for Comprehensive Research on Persons with Disabilities from the Japan Agency for Medical Research and Development (AMED) Grant Number 16dk0307029h0003 to MS.

## Conflict of Interest

The authors declare that the research was conducted in the absence of any commercial or financial relationships that may be construed as a potential conflict of interest.
